# Transabdominal Ultrasonography of the Small Bowel

**DOI:** 10.1155/2013/896704

**Published:** 2013-11-19

**Authors:** Rudolf Kralik, Peter Trnovsky, Marcela Kopáčová

**Affiliations:** ^1^GASTRO-SONOGRAFIA, s.r.o., Hviezdoslavova 23, 95701 Banovce nad Bebravou, Slovakia; ^2^Department of Internal Medicine of Hospital Banovce-3rd Private Hospital, Hviezdoslavova 23, 957 01 Banovce nad Bebravou, Slovakia; ^3^2nd Department of Medicine, Faculty of Medicine at Hradec Kralove, Charles University in Praha, University Teaching Hospital, Sokolska 581, 500 05 Hradec Kralove, Czech Republic

## Abstract

In the era of double balloon enteroscopy, capsule endoscopy, CT, and MRI enterography is transabdominal ultrasonography (TUS) underestimated method for evaluation of small bowel pathology. As often initial imagine method in abdominal complaints, nowadays has TUS much better diagnostic potential than two decades ago. High-resolution ultrasound probes with harmonic imaging significantly improve resolution of bowel wall in real time, with possibility to asses bowel peristalsis. Color flow doppler enables evaluation of intramural bowel vascularisation, pulse wave doppler helps to quantificate flow in coeliac and superior mesenteric arteries. Small intestine contrast ultrasonography with oral contrast fluid, as well as contrast enhanced ultrasonography with intravenous microbubble contrast also improves small bowel imaging. We present a review of small intestine pathology that should be detected during ultrasound examinations, discuss technical requirements, advantages and limitations of TUS, typical ultrasound signs of Crohn's disease, ileus, celiac disease, intussusception, infectious enteritis, tumours, ischemic and haemorrhagic conditions of small bowel. In the hands of experienced investigator, despite some significant limitations(obesity, meteorism), is transabdominal ultrasonography reliable, noninvasive and inexpensive alternative method to computerised tomography (CT) and magnetic resonance imaging (MRI) in small bowel examination.

## 1. Introduction

The reference diagnostic standard for all mucosal bowel diseases is endoscopy with histology, but some of small bowel diseases, despite introducing double balloon enteroscopy and capsule endoscopy, still need cross-sectional imaging, where nowadays dominate radiologic methods—CT enterography/enteroclysis and MRI enterography/enteroclysis. Whereas 20 years ago was diagnostic yield of bowel ultrasonography limited to detection of large tumours, ileus and extensive Crohn's disease, nowadays as one of the cross-sectional imaging methods transabdominal sonography has become established and relatively reliable method for examination of SB, thereby offers to gastroenterologists good possibility and reasons to amplify their diagnostic arsenal also in small bowel examination. 

Modern ultrasound devices with high-frequency (high resolution) probes and harmonic imaging significantly improve examination of SB by offering better overall image quality, better visualization of bowel pathology and associated changes in real time [1] (“live anatomy”). Wide availability, relatively low cost of modern devices, noninvasiveness, reproducibility, and absence of radiation make this diagnostic method “doctor and patient friendly”, enables frequently repeated examinations especially in chronic inflammatory small bowel diseases, and is safe also in young patients and pregnant women. Ultrasonographic examination provides correlation between clinical symptomatology and sonographic appearance of examined bowel segment (maximal tenderness, resistance, compressibility, presence or absence of peristalsis) [2] and gives to gastroenterologist other than only intraluminal view of bowel structures. However, sonography is highly operator dependent method and correct interpretation of sonographic findings needs adequate experience in abdominal and bowel sonography.

Spectrum of small bowel diseases reliably detectable by transabdominal ultrasonography now comprises Crohn's disease with all complications—strictures, fistulas, abscesses, tumours of proximal and distal part of SB, intussusceptions (owing to transient character often missed by CT and MRI), and ileus. In some conditions of SB (infectious enteritis, tuberculosis of SB, ischemic and haemorrhagic conditions of SB) can TUS contribute to correct diagnosis.

Using peroral (SICUS) and intravenous contrast (CEUS) offers images of SB pathology similar to the ones acquired by CT and MRI enterography, but reliable evaluation of entire small intestine by ultrasound is possible usually only in non-obese patients. However, advantage of high resolution sonography consist in high spatial resolution in pathological segment of SB, where focused TUS can provide additional information to CT and MRI imaging (especially in Crohn's disease).

In a meta-analysis of prospective studies comparing accuracy of CT, MRI, scintigraphy, PET, and TUS in inflammatory bowel disease (IBD) no significant differences were observed among these techniques—mean per-patient sensitivity (89.7%) and specificity (95.6%) and mean per-bowel segment sensitivity (92.9%) and specificity (92.9%) of TUS did not significantly differ from other evaluated methods [3].

High-resolution ultrasound probes (frequencies >7.5 Mhz) exhibit stratification of SB wall—with five different concentric layers—the first from the lumen is echogenic interface between lumen content and mucosa, then hypoechogenic mucosa, echogenic submucosa in the middle of wall, next hypoechogenic muscularis propria and the fifth—outer echogenic layer represents serosa and interface with perienteric structures. These sonographic layers practically correspond to histological layers [4]. Thickness of normal SB does not exceed 3 mm (with slight probe compression), stratification (five layers) is preserved, intramural vascularisation weak, peristalsis normal and lumen compressible.

High resolution (high-frequency) probes still have disadvantage of unsatisfactory penetration, so cannot be used in evaluating of deep abdominal structures, especially in obese patients, in addition, in some cases of initial forms of SB diseases false negative results are possible.

## 2. Technical Requirements for TUS of Small Bowel Examination

Reliability of sonographic examination depends on good-class ultrasound device with standard abdominal (2.5–6 Mhz) convex and high resolution linear or convex probe(7.5–14 MHz), both with harmonic mode, pulse wave doppler (PWD) for quantitative evaluation of celiac and superior mesentery flows, color flow doppler (CFD) and contrast enhanced ultrasonography (CEUS) software for detection and quantification of intramural vascularisation in thickened bowel wall and perienteric structures. Isosmotic polyethylene glycol (PEG) solution 1000 mL is required for small intestine contrast sonography (SICUS) [5]/enteroclysis [6] or hydrosonography [7] of SB. Second generation of microbubble contrast (e.g., SonoVue) 5–10 mL is needed for contrast enhanced sonography (CEUS) [8, 9].

Experienced sonographer with practice in abdominal and bowel ultrasonography, enough time (at least 30 min) for examination, and information about results of other imaging methods or surgery are also necessary for reliability of small bowel examination.

## 3. Technique of SB Examination 

Examination should be performed after overnight fasting, in supine position. 

In bowel examination we should use both standard (2.5–6 MHz) abdominal convex probe and high resolution (7.5–14 Mhz) probe [10].

Every examination of small bowel should be preceded by standard abdominal sonography with convex abdominal (2.5–6 Mhz) probe. This probe offers along with imaging of parenchymal abdominal organs also overall evaluation/panoramic view/of large and small bowel as well as flow parameters in coeliac (CA) and superior mesenteric (SMA) arteries.

Then examination with high resolution probe (7.5–14 Mhz) should be focused on the suspect pathological (thickened) segments of SB. This probe provides high spatial resolution but only in superficial structures (higher frequency = worse depth penetration). If a pathology is detected, wall thickness, stratification, luminal patency, degree of stenosis or dilatation, and motility pattern should be determined [10].

All parts of small bowel—duodenum, jejunum and ileum are accessible to TUS examination.

**Figure 1:**
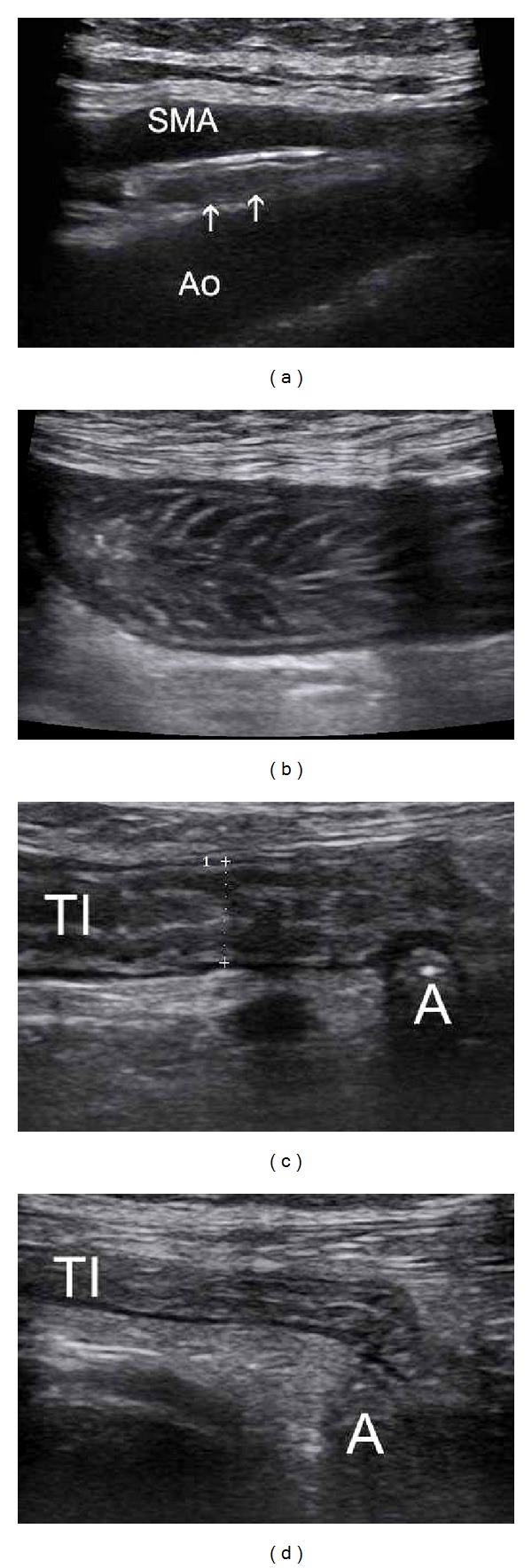
Normal small bowel: (a) Transverse view of pars horizontalis duodeni between aorta and SMA. (b) Longitudinal view of jejunum in left mesogastrium—with numerous valvulae conniventes. (c) Longitudinal section of terminal ileum (TI) in the left iliac fossa without and with compression by the probe (d). *A—transverse view of appendix. All with high resolution probe*.

Relatively stabile localisation of duodenum and terminal ileum (Figures 1(a), 1(b), 1(c), and 1(d)) makes these segments the best available for ultrasound imaging. Jejunum and nonterminal ileum due to length and variable localisation need systematic approach—we usually start examination with high-frequency probe in epigastric region by imaging of duodenum in transverse section, scanning it from duodenal bulb through descendant and horizontal parts of duodenum up to left epigastric-subcostal region (D4) Then systematically, scanning by parallel overlapping vertical or horizontal scanning lanes over all abdomen up to terminal ileum in the right lower quadrant. We use graded compression by the probe, which enables to evaluate compressibility, rigidity of bowel segments and to eliminate interference bowel gas.

*Small Intestine Contrast Ultrasonography (SICUS) or Hydrosonography*. TUS with using oral contrast solution (iso-osmolar nonabsorbable polyethylene glycol solution (PEG). The amount of PEG solution used in different studies varies between 200 and 2000 mL [5, 7, 11]. On average, the entire small intestine could be visualized on ultrasonography by about 45 min after the ingestion of 600 mL or less of contrast solution without any side effects [5] SICUS improves TUS resolution by separating of SB walls and eliminating bowel gas. Compared with conventional sonography luminal filling can improve visualisation of bowel walls and fold pattern [10], but extends time of examination (vary between 30–40 min). In the study of Pallotta et al. [12] diagnostic accuracy of SICUS is comparable to that of a radiologic examination, and is superior to that of standard TUS in detecting the presence, number, extension, and sites of small bowel lesions.

*Color Flow (Power) Doppler—CFD*. It is used to estimate presence, density or absence of vascular signals in thickened segments of bowel wall, in intraluminal or extraluminal pathological structures and for imaging flow in big abdominal vessels—SMA, coeliac trunk, portal vein. CFD is part of standard abdominal and bowel sonography.

*Duplex Scanning (TUS + PWD)*. B-mode assisted Pulse Wave Doppler can estimate flow parameters of coeliac trunk and SMA, usually with measurement of peak systolic velocity (PSV), end diastolic velocity (EDV), RI (resistance index = (PSV − EDV)/PSV), pulsatility index (PI) and minute flow volume (MFV) [13–16]. Quantification of flow by PWD in superior mesenteric artery should be standard part of bowel sonography.

In gastroenterological practice usually uses only PSV, EDV, RI, and MVF in SMA and CA.

*Triplex Scanning or Color Assisted Duplex Scanning (TUS + CFD + PWD)*. Enables evaluation of SMA/CA flow and intramural flow in thickened bowel segments.

*CEUS-Contrast Enhanced Ultrasonography.* is by EFSUMB recommendations [8] indicated only for evaluation of inflammatory activity in thickened bowel segments, discrimination between fibrous and inflammatory strictures in CD, and for discerning between abscesses and inflammatory infiltrates, and for confirming and following the route of fistula. CEUS must be preceded by TUS to set the localisation, extension of SB thickened segment and CFD for evaluation of intramural vascularisation.

 After standard TUS in CEUS specific harmonic mode we apply sulfur-hexafluoride based second-generation echo-signal enhancer (SonoVue) injected as a bolus 1.2–5 mL, folowed by 10 mL of isotonic saline, with watching enhancement of bowel wall in examined segment. Amount of 1.2 mL is usually sufficient with using standard abdominal probe in harmonic mode, high-frequency probes usually need higher amount of contrast. Every other examined segment needs another intravenous bolus of contrast. All CEUS examination shoud be videograbbed for analysis of enhancement patterns of each evaluated bowel segment, then by ultrasound device dedicated or PC software can be assessed the vascularisation of the examined bowel loop [8, 9, 17].

 Using CEUS can significantly extend time of examination, not only in real time, but also in analysing of videosequences of examination.

## 4. Transabdominal Ultrasonography in Crohn's Disease of Small Bowel

**Figure 2:**
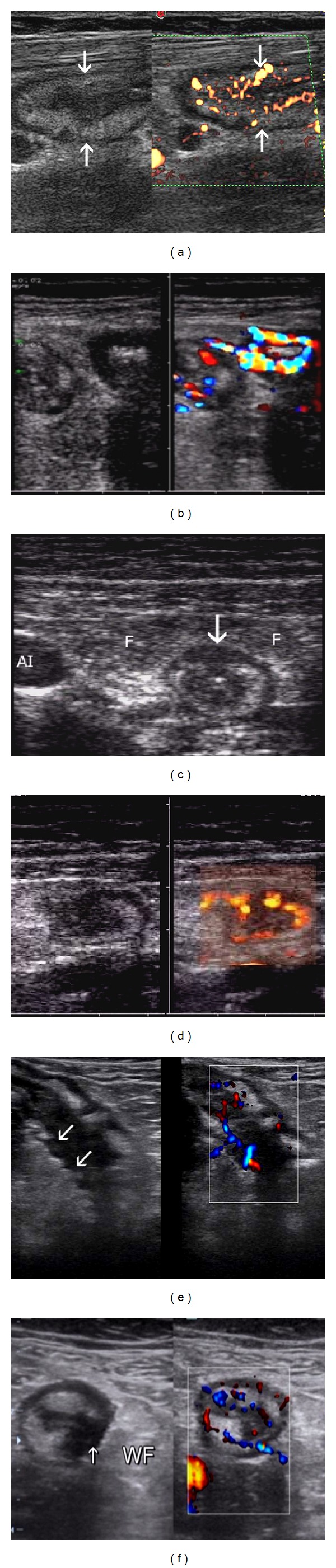
TUS in Crohn's disease. (a) Transversal view of thickened terminal ileum with preserved stratification and intramural hyper-vascularisation—*High resolution probe. *(b) Transversal section of two ileal bowel loops—proximal (left) with segmentally impaired stratification, distal with complete absence of stratification with hypoechogenic wall. The right half of picture shows intramural hyper-vascularisation especially in proximal loop, indicating active inflammation—*High resolution probe*. (c) Transversal view of terminal ileum with hypoechogenic bridge through echogenic submucosa between lumen and outer surface of the wall indicating transmural ulcer (arrow) and thickened inflamed “wrapping” fat (F)—*High resolution probe*. (d) Blind fistula wrapped by inflamed fat. Increased intramural vascularisation in color-Power Doppler (CFD)—*High resolution probe.* (e) Segmental absence of echogenic submucosa indicates longitudinal ulcer of terminal ileum (arrows) in longitudinal and (f) transversal view in a Crohn's ileitis (WF-inflamed fat)-FDsign—*High resolution probe. *

**Figure 3:**
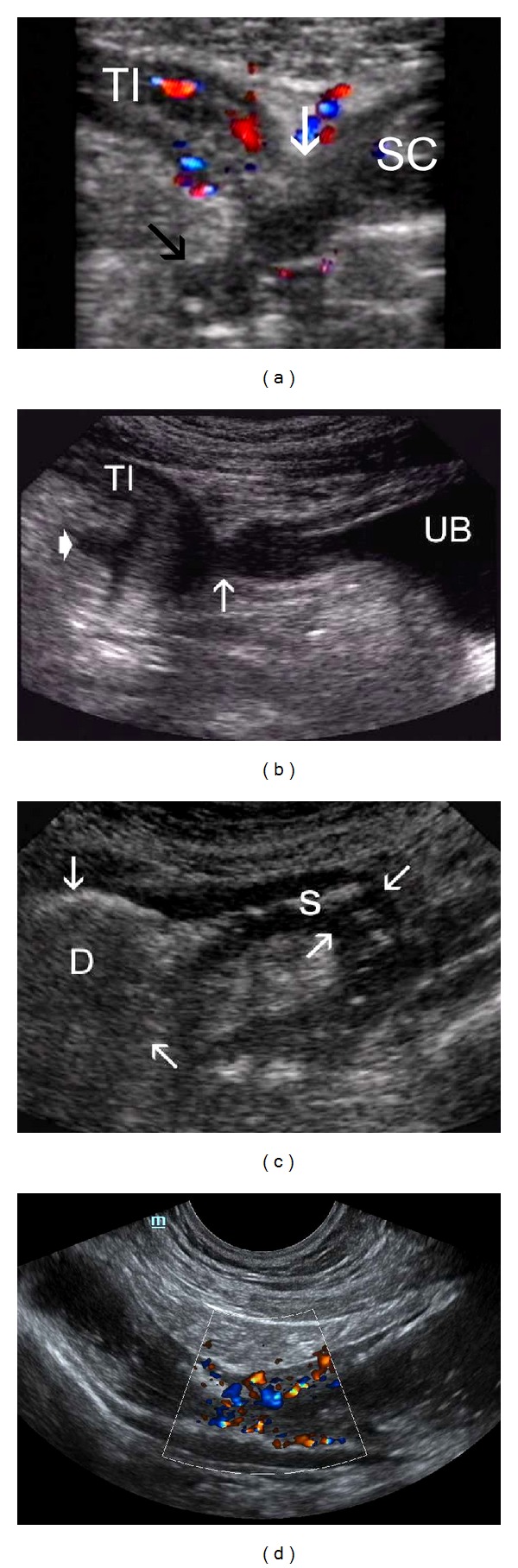
Crohn's disease complications. (a) Transverse view in lower abdomen shows fistula (white arrow) between terminal ileum/TI) and sigmoid colon (SC), black arrow points to small abscess, *high resolution probe*. (b) Oblique section of terminal ileum (TI) with blind fistula (thick arrow) into echogenic mesenterial fat and ileovesical fistula (thin arrow). *Standard abdominal probe*. (c) Stricture of ileum (S) with prestenotic dilatation (D)—*standard abdominal probe*. (d) TUS with color doppler and peroral contrast—Crohn's terminal ileum stenosis with intramural hypervascularisation (with CFD) indicates inflammatory stenosis—*high resolution probe. *

Crohn's disease (CD) of small bowel is usually suspected during initial TUS performed by experienced examiner. The basic sonographic feature of small bowel CD is segmentally thickened bowel wall (>3 mm) with or without preserved wall stratification, intramural vascularisation evaluated by CFD in active inflammation is usually high [18] (Figure 2(a), and 2(b)). Transmural character of inflammation offers wide spectrum of ultrasound pictures: transmural ulcerations (Figure 2(b)), longitudinal ulcers [19] (Figures 2(e) and 2(f)), with perienteric pathological changes—mesenteric and omental fat hypertrophy (“wrapping fat”) [20], blind fistulas (Figure 2(d)), enterocolic (Figure 3(a)), enterovesical fistulas (Figure 3(b)), abscesses [12, 17] (Figure 3(a)) and strictures [21–23] (Figures 3(c) and 3(d)). Numerous published articles evaluated the accuracy TUS with CFD, with or without peroral contrast (SICUS), in imaging the presence, activity, and complications of CD of SB, have confirmed high accuracy in detection of disease and its complications (fistulas, abscesses and stenoses), with good correlation with CT, MRI [3, 9, 21] and intraoperative findings [12, 21], but correlation with clinical CDAI has not been confirmed by all authors [9].

CEUS has potential of better intramural vascularisation imaging than CFD, so can be used to set the inflammatory activity in thickened bowel segments, to differentiate between inflammatory and fibrotic strictures, and between abscesses and infiltrates [7, 8, 15, 16, 18, 24], but is more time consuming, especially in multisegmental CD of SB.

TUS has also significant limitations in deep (pelvic) localised CD and in obese patients (insufficient penetration of high-frequency probes). Sufficient evaluation of TUS contribution in setting the diagnosis and evaluating stenosis, abscess, fistula, postoperative recurrence and activity of Crohn disease was recently documented by Calabrese et al. [23]. Need for frequent evaluation of Crohn's disease and thanks to absence of radiation exposure is TUS suitable especially in pediatric patients with Crohn disease and in pregnant women.

## 5. TUS in Celiac Disease

**Figure 4:**
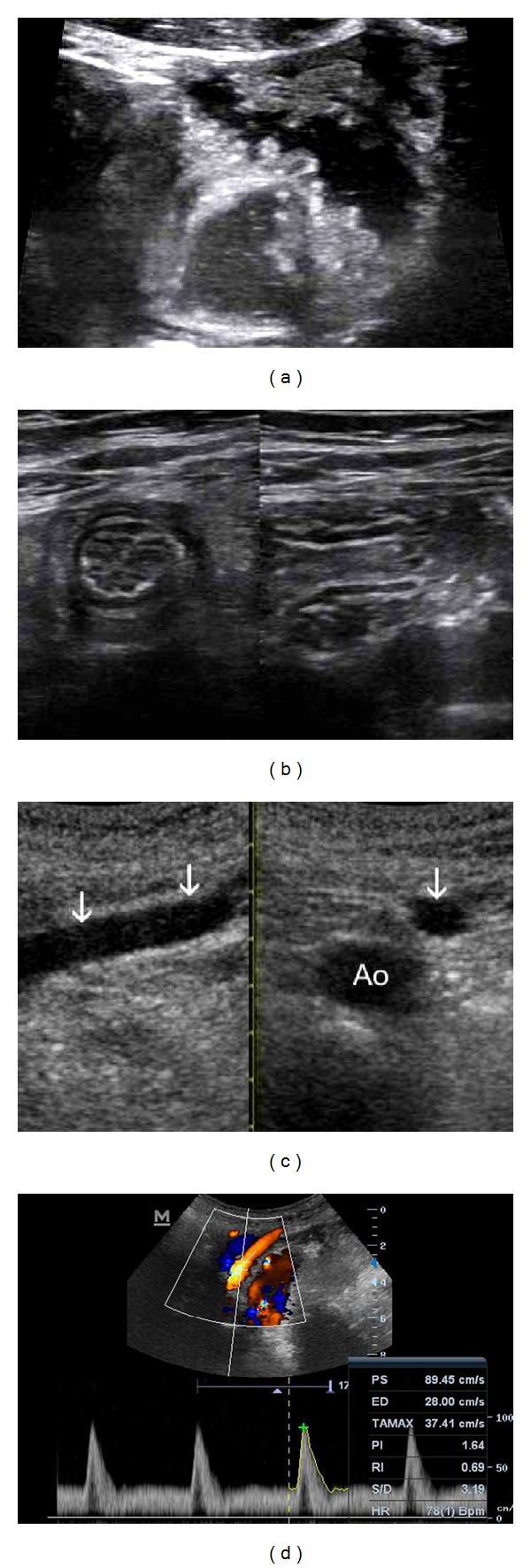
Celiac sprue: (a) Dilated loops of small bowel with thickened wall, and valvulae conniventes hyperperistalsis—*standard abdominal probe*. (b) Intussusception of jejunum in transverse (left) and longitudinal section in celiac sprue—*high resolution probe.* (c) Dilated SMA (9 mm) in a patient with untreated celiac disease—*standard probe*. (d) Low resistive index-RI (0.69) in SMA in untreated celiac disease—*standard probe. *

Despite the fact, that gold standard for the diagnosis of celiac disease is histologic confirmation of the intestinal damage in serologically positive individuals, in patients with untreated celiac disease we can regularly find out several sonographic signs that raise suspicion of this chronic disease also in clinically asymptomatic persons. Increased fluid content in moderately dilated bowel loops (25 to 35 mm) with hyperperistalsis in fasting state [25, 26], lightly thickened bowel wall (3–5 mm) and thickened valvulae conniventes (Figure 4(a)) [25, 27, 28] are most frequently seen in patients with untreated celiac sprue. Reduced number of jejunal folds and increase of ileal folds (jejunalisation of ileum) [27, 29], intermittent intussusceptions due to hyperperistalsis (Figure 4(b)), presence of slightly enlarged mesenterial lymph nodes (5–10 mm in short axis) [25–27, 29] and dilatation of SMA [25] with low resistive index [27] (Figure 4(c)) are also very frequent. In comparison to controls, celiac patients had higher superior mesenteric artery blood velocity and flow, with lower resistance indexes and higher portal vein velocity and flow in comparison to controls [30] (Figure 4(d)). Presence of small amount of free peritoneal fluid and increased gallbladder volume [26] are also seen in these patients.

None of the signs are specific, but combination of above mentioned signs is characteristic and indicates a suspicion of the disease [25].

## 6. TUS in Detection of Small Bowel Tumors

**Figure 5:**
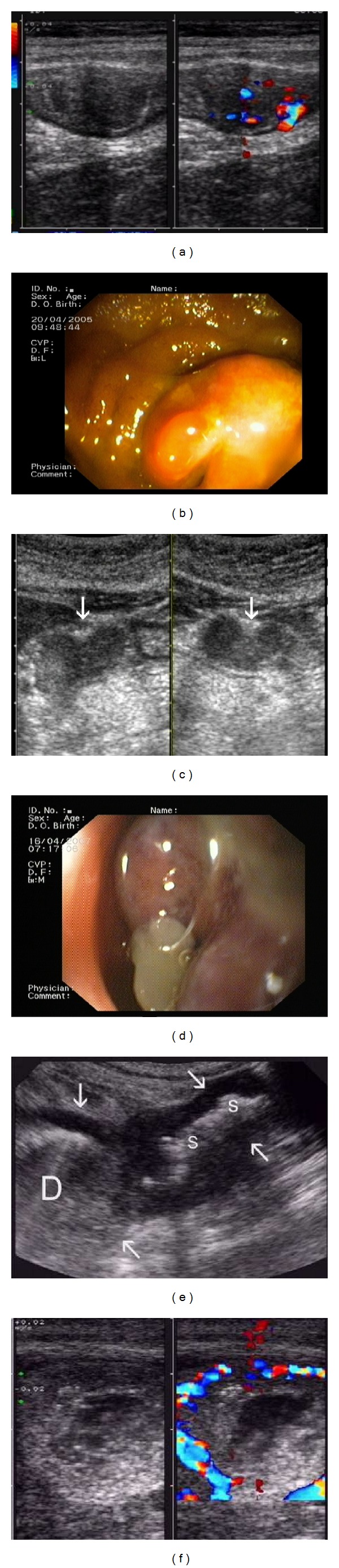
Tumors of small bowel. (a) Solid oval tumor in the lumen of terminal ileum with hypervascularisation in CFD (a) *High resolution probe*. (b) Endoscopic picture of tumor of terminal ileum in the same case-histologically carcinoid. (c) Oval solid tumor in D2 segment of duodenum—*Standard abdominal probe*. (d) Endoscopic view in the same case—histologically metastasis of Grawitz tumor (years after nephrectomy for tumor). (e) Longitudinal section of thickened small bowel loop (S) with stenosis and dilatation (D) of lumen. *Standard abdominal probe*. (f)Transversal view with *high resolution probe* in dilated segment shows hypervascularisation of thickened wall (f). Surgery confirmed suspected T-lymphoma of jejunum in untreated celiac disease.

The most frequently visualised tumors of SB are localised in duodenum and terminal ileum. Tumors in other parts of SB can be viewed after gaining significant volume and are causing clinical symptomatology. Among the malignant tumors are more frequent adenocarcinoma localised prevalently in duodenum, then carcinoids with prevalent localisation in terminal ileum, followed by lymphomas in ileum and jejunum, and less frequent mesenchymal tumors, predominantly in jejunum [31]. Most of the adenocarcinomas occurred in the duodenum and their relative frequency decreased in aboral direction: 29.9% in the jejunum and 16.0% in the ileum. The carcinoids showed an opposite trend, an increasing relative frequency in aboral direction: 3.9% in the duodenum, 9.2% in the jejunum and 86.7% in the ileum. Lymphomas were more frequent in the ileum (49.5%) compared to jejunum (29.4%) and duodenum (21.0%). Most sarcomas occurred along the jejunum (46.7%) [32]. Carcinoid tumors are oval hypoechogenic vascularised lesions (Figures 5(a) and 5(b)), lymphomas circularly affecting bowel segment with stenoses and dilatations of lumen [33] (Figures 5(e) and 5(f)). Most of gastro-intestinal lymphomas cause circumferential involvement of the bowel wall [34]. Metastatic tumors of SB (Figures 5(c) and 5(d)) as well as benign tumors are sporadically visualised by TUS due to intussusception caused by these tumors [35].

## 7. TUS in Vascular Problems of Small Bowel 

**Figure 6:**
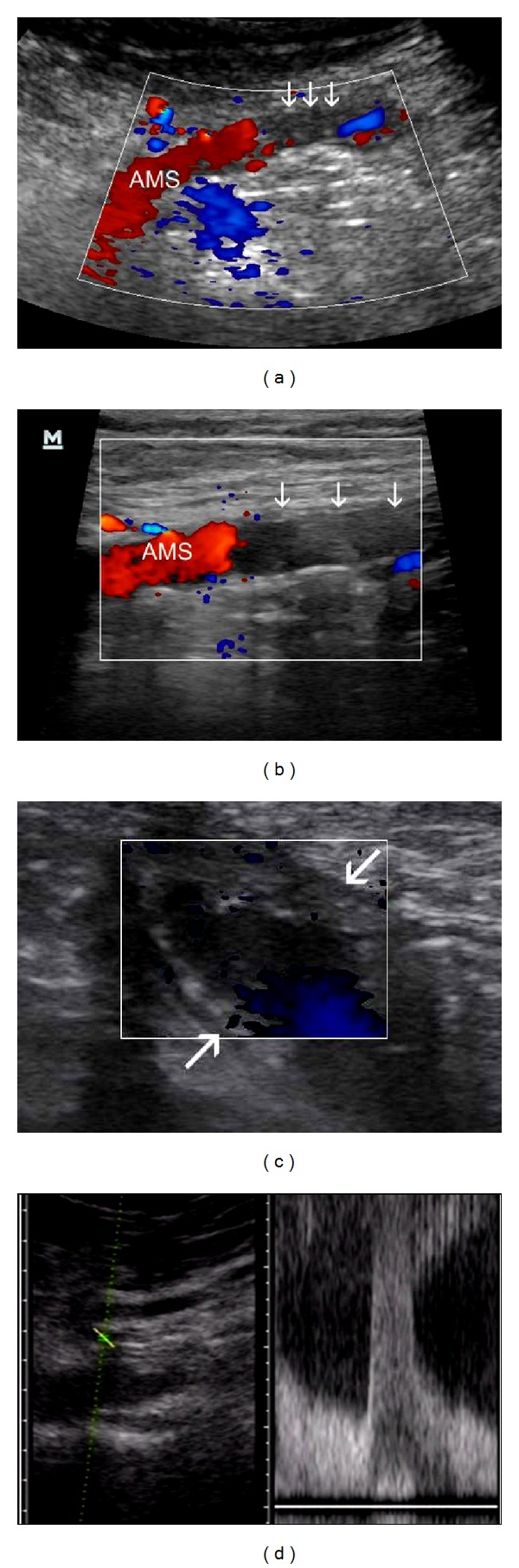
Vascular diseases of SB. (a) Fatal Thromboembolia of SMA in a patient with atrial fibrillation, with *standard abdominal probe*—absence of colour signal in embolised segment (arrows). (b) Use of *high resolution probe* in the same case. (c) Transversal view of jejunal loop without peristalsis, with thickened, avascular wall—another patient with SMA thromboembolia—but with presence of flow in proximal segment of SMA. *High resolution probe. *(e) Significant stenosis of SMA/Vmax over 400 cm/sek (>70%) in a patient with ischemic colitis. *Standard probe. *

The substantial part of SB is arterially supplied by superior mesenteric artery (SMA) except duodenum (part of celiac trunk). Imaging of celiac trunk and especially SMA should be done by all SB examinations, as well as evaluating of portal venous flow in accessible parts of portal vein. Absence of flow in SMA indicates occlusion (Figures 6(a), and 6(b)) and in an acute abdominal pain should be folowed by (CT) angiography. Ischemic bowel wall is in TUS typical thickened with the absence of CFD signals, lumen dilated (Figure 6(c)). High velocity of flow in superior mesenteric artery—SMA indicates significant stenosis (Figure 6(d)). PSV values can be used in detecting ≥50% and ≥70% SMA/CA stenosis: the peak systolic velocity PSV threshold that provided the highest overall accuracy (OA) for detecting ≥50% SMA stenosis was ≥295 cm/s (sensitivity 87%, specificity 89%, and OA 88%); and for detecting ≥70% SMA, it was ≥400 cm/s (sensitivity 72%, specificity 93%, and OA 85%) [16].

## 8. TUS in Small Bowel Ileus

**Figure 7:**
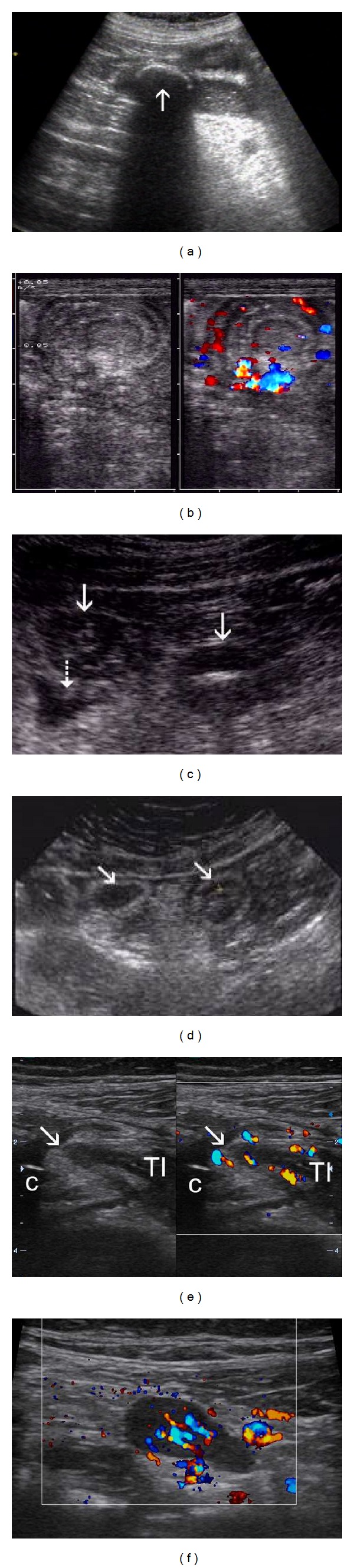
(a) Gallstone ileus—oval reflex with acoustic shadow in dilated jejunum. *Standard probe. *(b) Intussusception of jejunum. *High resolution probe. *(c) Spontaneous jejunal haematoma—transverse view of thickened hypoechogenic jejunal loops with absence of vascularization (in CFD)—arrows. Dotted arrow point to small peritoneal fluid. Patient in the hypocoagulation state, *high resolution probe. *(d) Spontaneous bowel haematoma transverse section of thickened jejunal loops with preserved stratification and narrowed lumen in a patient with hemophilia—(arrows)—*standard probe*. (e) longitudinal view of thickened terminal ileum (TI) and Bauhin's valve (arrow) with hypervascularisation of bowel wall (Yersinia ileocolitis)—*high resolution probe*. (f) Mesenterial lymphadenitis in the right iliac fossa in the same case—*high resolution probe*.

In a patient with typical symptomatology of ileus TUS shows dilated bowel loops with diameter usually above 35 mm, with stagnation of intraluminal fluid. In initial phase of this condition we can see hyperperistalsis of bowel loops, small amount of free peritoneal fluid between dilated bowel loops. In about 50% of cases we can find out cause of ileus (Figure 7(a)).

Truong et al. [36] in a retrospective trial investigated the significance of ultrasound in the diagnosis of intestinal obstruction in 459 patients. The overall sensitivity was 93.7%. In paralysis the correct diagnosis was obtained in 98% of all. Mechanical obstruction was identified in 91%. In cases of incomplete mechanical obstruction, sensitivity was 89%. The corresponding value for complete obstruction was 95%. In all patients with negative findings on abdominal X-ray (10%), the correct diagnosis was established by ultrasound. The underlying cause of ileus was yielded by ultrasound in 45% of the cases. Ultrasound is proven to be of significant importance in the diagnosis and differentiation of ileus.

Ultrasound may detect the cause of ileus with specific sonographic findings such as external hernias, intestinal intussusception, tumors, ascariasis, superior mesenteric artery syndrome, bezoars, foreign bodies, and Crohn's disease.

Sonographic findings suggesting a need for surgery include intraperitoneal free fluid, bowel wall thickness of more than 4 mm, and decreased or absent peristalsis in previously documented mechanically obstructed bowel. Bowel wall perfusion can be assessed by color doppler sonography, and the presence of free intraperitoneal air indicates bowel perforation [37]. CT scan can detect up to 100% of complete and incomplete SB obstruction and its cause [38], and so it should be preferred in cases with unclear TUS findings. 

## 9. TUS in Detection of Small Bowel Haematomas

Haematomas of SB are usually sporadic complication of hypocoagulation states—especially caused by anticoagulation pharmacotherapy. In ultrasound view are small bowel haematomas typical with segmentally concentrically thickened bowel wall with or without preserved stratification (Figures 7(c) and 7(d)) and with minimal or absent intramural vascularisation in CFD. Lumen of affected bowel segment is stenotic (anticoagulant ileus) what corresponds with complaints of patient (ileus symptomatology) [39, 40]. CT or MRI is needed in equivocal TUS findings in patients with hypocoagulation conditions [39].

## 10. TUS in Intussusception of Small Bowel

Intussusception (invagination) of SB is in TUS typical by multilayered structure with onion or donut appearance in transverse view (Figure 7(b)). In adult population are intussusceptions sporadically incidentally seen during abdominal TUS, or in SB inflammations, celiac disease, tumors of SB. Frequently are self-limiting, idiopathic or related to celiac or Crohn's disease, in about 25% are asymptomatic [41], however some can hide benign or malignant or metastatic tumors [35, 42]. Other imaging methods (CT, MRI) are indicated in suspicion of tumour(s).

## 11. TUS in Infectious Enteritis and Enterocolitis 

Sonography in acute enteritis shows thickened inflamed bowel wall, usually with preserved stratification and with intramural hypervascularisation (in colour doppler) and hyperperistalsis. In some cases, especially caused by Yersinia enterocolitica, Campylobacter jejuni and Salmonella enteritidis [43] significantly thickened terminal ileum and caecum in right lower quadrant along with mesenterial lymphadenitis can mimic Crohn's disease or acute appendicitis (Figures 7(e) and 7(f)). Owing to usually transient character of these conditions are other imaging methods not necessary.

## 12. TUS in Small Bowel Tuberculosis and Whipple's Disease

Transabdominal ultrasonography in 66 patients with abdominal tuberculosis [43] revealed ascites (56%), lymphadenopathy (18%), intestinal involvement (8%), and mesenteric abscesses and thickened omentum only in 3% of patients. Barreiros et al. [44] in a group of 7 patients with intestinal tuberculosis sonographically, asymmetric thickening of small bowel wall (in 100% patients), intramural abscesses (86%), fistulas (43%), mesenteric thickening and white bowel sign (both 29%), enlarged mesenterial lymph nodes with inhomogenous echostructure and hypoechogenic spots (86%), and ascites (29%) were detected. Hollerweger and Dietrich [45] introduced the term “white bowel” in cases of hyperechoic appearance of thickened bowel wall seen sonographically in 10 patients, with Whipple's disease (*n* = 2), Mycobacterium avium intracellulare infection (*n* = 3), T-cell lymphoma (*n* = 2) and in carcinoma of small and large intestine (*n* = 3), and most patient had enlarged lymph nodes and so this phenomenon was very probably caused by lymph oedema of small bowel wall.

## 13. Perspectives of TUS in Small Bowel Imaging 

 Transcutaneous ultrasound elasticity imaging (UEI) is a promising, noninvasive approach for measuring tissue mechanical properties, that can differentiate inflammatory from fibrotic intestine in rat models of IBD and can differentiate between fibrotic and unaffected intestine in humans with CD [46]. Promising results of the study about diagnostic performance of 3-dimensional ultrasound of small bowel with using tap water as oral contrast material [47] might also strengthen the position of TUS among SB imaging methods.

## 14. Conclusion 

TUS as, usually, the first diagnostic procedure in abdominal complaints reveals the most of Crohn's SB inflammations, ileus, and intussusceptions enable to express suspicion of celiac disease, significant stenosis, or SMA occlusion. In the known Crohn's disease transabdominal ultrasonography with oral contrast, color doppler, and in some cases intravenous contrast can reliably evaluate segmental inflammatory activity, local, and distal complications of a disease. Thanks to noninvasiveness and lack of radiation, TUS is a relatively good alternative to CT or MRI enterography, particularly in young patients and pregnant women. In the duodenum and terminal ileum, TUS can detect the most of benign and malignant tumors. TUS is patient and doctor friendly, noninvasive, and low-cost diagnostic procedure, and despite some significant limitations (obesity, meteorism), in the hands of experienced examiner offers reliable tool for SB diseases examination.

## List of Abbreviations

CDAI: Crohn's disease activity index
CEUS: contrast enhanced ultrasonography
CFD: colour flow doppler
CT: computerised tomography
CD: Crohn's Disease
EDV: end diastolic velocity
IBD: inflammatory bowel disease
MFV: minute flow volume
MRI: magnetic resonance imaging
PI: pulsatility index
PWD: pulse wave doppler
PSV: peak systolic velocity
RI: resistance index
SICUS: small intestine contrast ultrasonography
SMA: superior mesenteric artery
TUS: transabdominal ultrasonography.

## References

[1] Schmidt T, Hohl C, Haage P (2005). Phase-inversion tissue harmonic imaging compared to fundamental B-mode ultrasound in the evaluation of the pathology of large and small bowel. *European Radiology*.

[2] Kuzmich S, Howlett DC, Andi A, Shah D, Kuzmich T (2009). Transabdominal sonography in assessment of the bowel in adults. *American Journal of Roentgenology*.

[3] Horsthuis K, Bipat S, Bennink RJ, Stoker J (2008). Inflammatory bowel disease diagnosed with US, MR, scintigraphy, and CT: meta-analysis of prospective studies. *Radiology*.

[4] Kimmey MB, Martin RW, Haggitt RC, Wang KY, Franklin DW, Silverstein FE (1989). Histologic correlates of gastrointestinal ultrasound images. *Gastroenterology*.

[5] Pallotta N, Baccini F, Corazziari E (2000). Small intestine contrast ultrasonography. *Journal of Ultrasound in Medicine*.

[6] Nagi B, Rana SS, Kochhar R, Bhasin DK (2006). Sonoenteroclysis: a new technique for the diagnosis of small bowel diseases. *Abdominal Imaging*.

[7] Folvik G, Bjerke-Larssen T, Ødegaard S, Hausken T, Gilja OH, Berstad A (1999). Hydrosonography of the small intestine: comparison with radiologic barium study. *Scandinavian Journal of Gastroenterology*.

[8] Piscaglia F, Nolsøe C, Dietrich CF (2012). The EFSUMB guidelines and recommendations on the clinical practice of contrast enhanced ultrasound (CEUS): update 2011 on non-hepatic applications. *Ultraschall in der Medizin*.

[9] Malagò R, D’Onofrio M, Mantovani W (2012). Contrast-enhanced ultrasonography (CEUS) vs. MRI of the small bowel in the evaluation of Crohn’s disease activity. *La Radiologia Medica*.

[10] Nylund K, Ødegaard S, Hausken T (2009). Sonography of the small intestine. *World Journal of Gastroenterology*.

[11] Mirk P, Foschi R, Minordi LM (2011). Sonography of the small bowel after oral administration of fluid: an assessment of the diagnostic value of the technique. *Radiologia Medica*.

[12] Pallotta N, Vincoli G, Montesani C (2012). Small intestine contrast ultrasonography (SICUS) for the detection of small bowel complications in crohn’s disease: a prospective comparative study versus intraoperative findings. *Inflammatory Bowel Diseases*.

[13] Mitchell EL, Moneta GL (2006). Mesenteric duplex scanning. *Perspectives in Vascular Surgery and Endovascular Therapy*.

[14] Nakamura T, Moriyasu F, Ban N (1989). Quantitative measurement of abdominal arterial flow using image-directed Doppler ultrasonography: superior mesenteric, splenic, and common hepatic arterial blood flow in normal adults. *Journal of Clinical Ultrasound*.

[15] Gentile AT, Moneta GL, Lee RW, Masser PA, Taylor LM, Porter JM (1995). Usefulness of fasting and postprandial duplex ultrasound examinations for predicting high-grade superior mesenteric artery stenosis. *The American Journal of Surgery*.

[16] Aburahma AF, Stone PA, Srivastava M (2012). Mesenteric/celiac duplex ultrasound interpretation criteria revisited. *Journal of Vascular Surgery*.

[17] Ripolles T, Martinez-Perez MJ, Blanc E (2011). Contrast-enhanced ultrasound (CEUS) in Crohn’s disease: technique, image interpretation and clinical applications. *Insights into Imaging*.

[18] Spalinger J, Patriquin H, Miron M-C (2000). Doppler US in patients with Crohn disease: vessel density in the diseased bowel reflects disease activity. *Radiology*.

[19] Kunihiro K, Hata J, Haruma K, Manabe N, Tanaka S, Chayama K (2004). Sonographic detection of longitudinal ulcers in Crohn disease. *Scandinavian Journal of Gastroenterology*.

[20] Maconi G, Greco S, Duca P (2008). Prevalence and clinical significance of sonographic evidence of mesenteric fat alterations in Crohn’s disease. *Inflammatory Bowel Diseases*.

[21] Parente F, Bianchi Porro G, Maconi G (2002). Bowel ultrasound in assessment of Crohn’s disease and detection of related small bowel strictures: a prospective comparative study versus X ray and intraoperative findings. *Gut*.

[22] Panés J, Bouzas R, Chaparro M (2011). Systematic review: the use of ultrasonography, computed tomography and magnetic resonance imaging for the diagnosis, assessment of activity and abdominal complications of Crohn’s disease. *Alimentary Pharmacology and Therapeutics*.

[23] Calabrese E, Zorzi F, Zuzzi S (2012). Development of a numerical index quantitating small bowel damage as detected by ultrasonography in Crohn’s disease. *Journal of Crohn’s and Colitis*.

[24] Sjekavica I, Barbarić-Babić V, Krznarić Ž, Molnar M, Čuković-Čavka S, Štern-Padovan R (2007). Assessment of Crohn’s disease activity by Doppler ultrasound of superior mesenteric artery and mural arteries in thickened bowel wall: cross-sectional study. *Croatian Medical Journal*.

[25] Rettenbacher T, Hollerweger A, Macheiner P, Huber S, Gritzmann N (1999). Adult celiac disease: US signs. *Radiology*.

[26] Fraquelli M, Colli A, Colucci A (2004). Accuracy of ultrasonography in predicting celiac disease. *Archives of Internal Medicine*.

[27] Dell’Aquila P, Pietrini L, Barone M (2005). Small intestinal contrast ultrasonography-based scoring system: a promising approach for the diagnosis and follow-up of celiac disease. *Journal of Clinical Gastroenterology*.

[28] Castiglione F, Rispo A, Cozzolino A (2007). Bowel sonography in adult celiac disease: diagnostic accuracy and ultrasonographic features. *Abdominal Imaging*.

[29] Dietrich CF, Brunner V, Seifert H, Schreiber-Dietrich D, Caspary WF, Lembcke B (1999). Intestinal B-mode sonography in patients with endemic sprue. Intestinal sonography in endemic sprue. *Ultraschall in der Medizin*.

[30] Magalotti D, Volta U, Bonfiglioli A, Ramilli S, Berzigotti A, Zoli M (2003). Splanchnic haemodynamics in patients with coeliac disease: effects of a gluten-free diet. *Digestive and Liver Disease*.

[31] Neugut AI, Jacobson JS, Suh S, Mukherjee R, Arber N (1998). The epidemiology of cancer of the small bowel. *Cancer Epidemiology Biomarkers and Prevention*.

[32] Gabos S, Berkel J, Band P, Robson D, Whittaker H (1993). Small bowel cancer in Western Canada. *International Journal of Epidemiology*.

[33] Rioux M, Langis P, Naud F (1995). Sonographic appearance of primary small bowel carcinoid tumor. *Abdominal Imaging*.

[34] Goerg C, Schwerk WB, Goerg K (1990). Gastrointestinal lymphoma: sonographic findings in 54 patients. *American Journal of Roentgenology*.

[35] Aissa A, Kherifech M, Alouini R, Hajji H, Stita W (2012). Multiple intussusceptions revealing metastases from renal carcinoma to the small intestine. *Journal of Visceral Surgery*.

[36] Truong S, Arlt G, Pfingsten F, Schumpelick V (1992). The significance of sonography in the diagnosis of ileus. A retrospective study in 459 patients. *Der Chirurg; Zeitschrift für alle Gebiete der operativen Medizen*.

[37] Hefny AF, Corr P, Abu-Zidan FM (2012). The role of ultrasound in the management of intestinal obstruction. *Journal of Emergencies, Trauma and Shock*.

[38] Frager D, Medwid SW, Baer JW, Mollinelli B, Friedman M (1994). CT of small-bowel obstruction: value in establishing the diagnosis and determining the degree and cause. *American Journal of Roentgenology*.

[39] Abbas MA, Collins JM, Olden KW (2002). Spontaneous intramural small-bowel hematoma: imaging findings and outcome. *American Journal of Roentgenology*.

[40] Chaiteerakij R, Treeprasertsuk S, Mahachai V, Kullavanijaya P (2008). Anticoagulant-induced intramural intestinal hematoma: report of three cases and literature review. *Journal of the Medical Association of Thailand*.

[41] Maconi G, Radice E, Greco S, Bezzio C, Bianchi Porro G (2007). Transient small-bowel intussusceptions in adults: significance of ultrasonographic detection. *Clinical Radiology*.

[42] Butte JM, Meneses M, Waugh E, Parada H, De La Fuente H (2009). Ileal intussusception secondary to small bowel metastases from melanoma. *The American Journal of Surgery*.

[43] Malik A, Saxena NC (2003). Ultrasound in abdominal tuberculosis. *Abdominal Imaging*.

[44] Barreiros AP, Braden B, Schieferstein-Knauer C, Ignee A, Dietrich CF (2008). Characteristics of intestinal tuberculosis in ultrasonographic techniques. *Scandinavian Journal of Gastroenterology*.

[45] Hollerweger A, Dietrich CF (2005). ‘White bowel’. A sonographic sign of intestinal lymph edema?. *Ultraschall in der Medizin*.

[46] Stidham RW, Xu J, Johnson LA (2011). Ultrasound elasticity imaging for detecting intestinal fibrosis and inflammation in rats and humans with Crohn’s disease. *Gastroenterology*.

[47] Elwagdy S, Ramadan MR, Farag MA (2011). Diagnostic performance of ultrafast 3-dimensional ultrasound on the small bowel: single center experience. *Clinical Medicine Insights: Gastroenterology*.

